# Bulk pollen sequencing reveals rapid evolution of segregation distortion in the male germline of Arabidopsis hybrids

**DOI:** 10.1002/evl3.96

**Published:** 2019-01-30

**Authors:** Russell Corbett‐Detig, Paloma Medina, Hélène Frérot, Christelle Blassiau, Vincent Castric

**Affiliations:** ^1^ Genomics Institute and Department of Biomolecular Engineering UC Santa Cruz Santa Cruz California 95064; ^2^ Université de Lille CNRS UMR 8198‐Evo‐Eco‐Paleo F‐59000 Lille France

**Keywords:** Arabidopsis, hybridization, segregation distortion, speciation

## Abstract

Genes that do not segregate in heterozygotes at Mendelian ratios are a potentially important evolutionary force in natural populations. Although the impacts of segregation distortion are widely appreciated, we have little quantitative understanding about how often these loci arise and fix within lineages. Here, we develop a statistical approach for detecting segregation distorting genes from the comprehensive comparison of whole genome sequence data obtained from bulk gamete versus somatic tissues. Our approach enables estimation of map positions and confidence intervals, and quantification of effect sizes of segregation distorters. We apply our method to the pollen of two interspecific F1 hybrids of *Arabidopsis lyrata* and *A. halleri* and we identify three loci across eight chromosomes showing significant evidence of segregation distortion in both pollen samples. Based on this, we estimate that novel segregation distortion elements evolve and achieve high frequencies within lineages at a rate of approximately one per 244,000 years. Furthermore, we estimate that haploid‐acting segregation distortion may contribute between 10% and 30% of reduced pollen viability in F1 individuals. Our results indicate haploid acting factors evolve rapidly and dramatically influence segregation in F1 hybrid individuals.

**Impact Summary**

We often think of natural selection as acting exclusively to shape the genomes of adult diploid individuals. However, this neglects the potentially dramatic impacts of genes that maximize their fitness by either distorting the basic fairness of the meiotic process or by engaging in haploid selection during the sperm or egg phases, ultimately segregating into viable gametes at higher than the expected 50:50 Mendelian ratio. A sperm or pollen gene that maximizes its chances for fertilizing eggs, even to the detriment of the diploid adult, can be favored by natural selection. However, because segregation distorters are often challenging to detect, their prevalence within and between species and their rate of evolution is largely unknown. In this work, we develop a sequencing and analysis approach for accurately detecting and fine‐mapping even small effect segregation distortion genes. Using *Arabidopsis* hybrids, we show that three loci distort segregation in the pollen genome suggesting the rate of evolution of these genes is rapid. This approach will enable more quantitative and unbiased surveys of segregation distortion in diverse model and nonmodel organisms than has previously been possible.

The unbiased segregation of alleles through heterozygous individuals—Mendelian Inheritance—is a fundamental tenet of genetics. Nonetheless, some genetic elements subvert this basic fairness by either biasing the meiotic segregation process itself (meiotic drive) or by altering the fitness of haploid germline cells (haploid selection) such that traditional Mendelian ratios are not recovered in subsequent generations. These genetic elements—collectively referred to here as segregation distorters—are substantially less well understood than factors influencing the fitness of diploid individuals. Nonetheless, the evolution of segregation distortion elements may have profound evolutionary implications (Novitski et al. 1962; Burt and Trivers 2009; Lindholm et al. 2016).

One type of segregation distorter actively influences gamete genotype ratios by disable or destroying competitor gametes that do not inherit the same allele. These “selfish genes” can rise in frequency and fix within populations, even if they do not confer an advantage to their carriers, by simply ensuring successful transmission (Sandler and Novitski 1957; Novitski et al. 1962). Although initially thought to be genetic curiosities, segregation distorters have been documented in numerous species (Taylor and Ingvarsson 2003; Burt and Trivers 2009; Lindholm et al. 2016). Furthermore, interspecific crosses often reveal such genetic systems that have fixed within populations, and which sometimes contribute to reproductive isolation (Fishman and Willis 2005; Phadnis and Orr 2009). However, because the phenotypic consequences of segregation distorters in heterozygous individuals are often subtle, and because classical methods for detecting these genes require large effect sizes, the frequencies of segregation distorters in natural populations and their rate of evolution between divergent taxa remain largely unknown (Taylor and Ingvarsson 2003; Burt and Trivers 2009).

Transmission ratio distortion (TRD) is a powerful method to identify non‐Mendelian segregation in a controlled cross (Fishman and Willis 2005; Leppala et al. 2013). Briefly, a TRD experiment includes (1) crossing individuals from two partially isolated lineages, (2) intercrossing their F1 progeny, (3) raising a large population of F2 individuals, and (4) genotyping F2's at markers distributed genome‐wide. Loci whose genotype ratios deviate significantly from Mendelian expectations are then candidate regions containing segregation distorerse and genetic elements contributing to reproductive isolation. For example, genes that contribute to viability differences among F2 hybrids can be detected as a systematic skew toward one ancestry type surrounding that locus. In addition, if male gametes are differentially able to develop and successfully fertilize the female germline, the F2 ancestry ratios can reflect such gametic competition as well.

Despite the popularity of TRD‐based analyses, there are many drawbacks for studies specifically aimed at surveying precisely for segregation distortion (Corbett‐Detig et al. 2015b). First, due in large part to practical constraints associated with raising large cohorts of F2 progeny, most TRD analyses have little power to detect loci with small effect sizes and consequently mapping confidence intervals tend to be quite broad. Second, because TRD is not assayed until after the F2's have grown, it is usually infeasible to distinguish between genes acting during the gametic phase and those acting during the zygotic phase of an organism's lifecycle (although see (Leppala et al. 2013) for a statistical approach for distinguishing gametic and zygotic effects). Therefore, comprehensively identifying, quantifying, and surveying natural populations for segregation distortion occurring during the haploid phase is an important challenge in evolutionary genomics.

Directly sequencing pools of gametes, rather than individuals from F2 populations offers appealing insights into the genetic basis of non‐Mendelian inheritance that TRD does not ((Corbett‐Detig et al. 2015b; Larson et al. 2018) see also (Bélanger et al. 2016; Wei et al. 2017) for related applications). For many species, it is infeasible to generate sufficient F2 individuals to enable powerful analyses of TRD. However, the amount of male germ cells that can be obtained for a given individual is often virtually unlimited across a wide array of organisms. Therefore, gamete sequencing is an appealing means to study haploid‐acting segregation distortion for two primary reasons. First, because each sperm or pollen cell is essentially an independent meiotic event, our power to identify distorting genes is limited by sequencing depth rather than the number of informative progeny. Second, because gametes are sampled before they form zygotes and develop, this approach removes the impact of viability and gametic competition. These features make sequencing gamete pools a particularly appealing method to identify and to precisely quantify the impacts of segregation distortion elements.

*Arabidopsis lyrata* and *A. halleri* are two recently diverged species that are in the early phases of reproductive isolation (Roux et al. 2011; Wang et al. 2010) and interspecific hybrids can be formed in the lab (de Meaux et al. 2006; Willems et al. 2007). Despite numerous genomic regions showing strong TRD in a backcross (Willems et al. 2007) and a F2 population (Frérot et al. 2010) between *A. lyrata* and *A. halleri*, little is known about the genetic basis of this trait. Furthermore, although *A. lyrata* and *A. halleri* are partially genetically isolated, they share abundant genetic polymorphisms and in particular S‐haplogroups, indicating they are or have recently been capable of hybridizing and exchanging genes in natural populations (Castric et al. 2008, 2010; Novikova et al. 2016). Finally, *A. lyrata* has a published high‐quality reference genome (Hu et al. 2011). Hybrids of *A. lyrata* and *A. halleri* are therefore an appealing system for quantifying the impacts of segregation distortion in hybrid individuals.

Here, we develop a powerful maximum likelihood‐based approach to estimate the effect size and genomic positions of loci that distort segregation in F1 males. By applying our method to sequence data from somatic tissue and bulk pollen from two *A. lyrata*/*A. halleri* F1 hybrid individuals, we identify and map three candidate haploid‐acting segregation distortion loci. We estimate the effect sizes of these loci and discuss the implications of our findings and of our approach for understanding the evolution of segregation distortion and speciation among diverse lineages.

## Methods

### PLANT HUSBANDRY AND CROSSES

F1 individuals were obtained for two independent controlled crosses by depositing *A. halleri* pollen on *A. lyrata* pistils. The parents used for the two crosses were distinct. The two *A. halleri* parents were from closely related Italian populations (I14 and I16 in (Frérot et al. 2017)) and the *A. lyrata* parents were distinct individuals from a single population in Central Bohemia (Czech Republic (Macnair et al. 1999); Table S1). Seeds from the reciprocal crosses did not germinate. Plants were vernalized for 8 weeks and brought to flowering under natural light conditions at 19°C in the greenhouse. For each cross we chose one F1 plant with abundant flower production to proceed with pollen isolation.

### LIBRARY PREPARATION AND SEQUENCING

For each F1 plant, pollen was isolated from 100–500 flowers that were collected in 50% EtOH and stored at –20°C in Falcon tubes. The Falcon tubes were gently vortexed to detach pollen from anthers. Flowers and eventual flower debris were manually removed and the ethanol with pollen in suspension was transferred to clean tubes for centrifugation at 13,000 rpm for 10 minutes. The pollen pellet was dried at room temperature for 1h and visually inspected under the microscope to confirm absence of remaining debris of somatic tissues. DNA was then extracted using the Macherey Nagel Nucleospin food kit with columns from the Tissu XS kit from the same provider. Libraries were constructed using a Nextera library preparation kit. We deeply sequenced all libraries (two *A. lyrata* parents, two *A. halleri* parents, two F1 offspring somatic and germline (pollen) samples) on two lanes of HiSeq4000 using 100 bp paired‐end reads at the UCB Vincent J. Coates sequencing center.

### SHORT READ PROCESSING AND ALIGNMENT

We first sought to identify the subset of single nucleotide polymorphisms (SNPs) that consistently distinguished the members of each species. To do this, we aligned all short read data from each individual to the *A. lyrata* reference genome (Hu et al. 2011) using the mem function of BWA v0.7.15‐r1140 (Li and Durbin 2009). We then used the Genome Analysis Toolkit v3.4.46 (DePristo et al. 2011; McKenna et al. 2010) to realign indels and we genotyped each individual using the “HaplotypeCaller” function. We retained variant sites with a genotype quality of 30 of higher, and required that each site be fixed between the parental species and a heterozygote in both F1 somatic samples. To mitigate against the impacts of genome structural variants, which might confound allele frequency‐based analyses in pools of gametes, we removed the subset of sites below 220 or above 400 total coverage across all libraries. These values were selected as the 10% and 90% quantile of the empirical site depth distribution. Visual inspection of the depth distribution (Fig. S1), confirmed that these cutoffs were sufficient to remove the majority of sequencing depth outlier sites.

To reduce the impacts of biased mapping between somatic and germline libraries, which could produce strongly skewed ancestry ratios, we mapped only the first read in each pair and subsampled the read length distributions of reads in each library to be exactly identical between somatic and germline samples after trimming adapter sequences using Trimmomatic v0.32 (Bolger et al. 2014). Scripts to perform these trimming functions are provided in the GitHub repository associated with this project (https://github.com/russcd/MAP_SD). Finally, we discarded ancestry informative sites that are within 100 bp of one another to preclude the possibility that single reads would be counted twice. Then, we counted the number of alleles of each type at each site that we retained as ancestry informative in comparing the parental genomes (above).

### MAXIMUM LIKELIHOOD ESTIMATION OF EFFECT SIZES AND DISTORTING LOCI MAP POSITIONS

Because of ongoing recombination during meiosis, the locations of elements that distort segregation in the germline can be mapped by comparing ancestry ratios from libraries made from germline and somatic tissue (Corbett‐Detig et al. 2015b; Wei et al. 2017). Additionally, the relative skew in the ancestry ratios between reads derived from somatic and germline tissue at a site of a distorting element is expected to be proportional to the effect size of a distorting locus. Whereas previous efforts have sought to test for segregation distortion within individual genomic windows, essentially all sites on a distorted chromosome contain information about the effect and location of distorting elements due to linkage. We therefore sought to develop a simple maximum likelihood‐based approach that leverages chromosome‐wide allele count information for estimating both the position and the effect size of a candidate distorter and for quantifying our uncertainty in the estimated position by constructing mapping confidence intervals.

For a given candidate distorting locus at position i, we seek to estimate the segregation ratio, k, by optimizing the likelihood of the germline short read data mapped onto the same chromosome conditional on i and k. That is, for a site containing an ancestry informative allele, p, the distance to the distorting locus, i, in basepairs is |i–p|. This can be converted to distance in Morgans using a recombination map if available. Then, we apply Haldane's mapping function and convert the recombinational distance to the probability that a recombination event has occurred somewhere in the interval between the distorting element and the marker (i,p), which we term r_ip_. Here, we use the high density recombination map of (Hämälä et al. 2017), to convert between the physical positions and recombinational distance for all markers using the piecewise approach of (Corbett‐Detig et al. 2015a) to fit a smooth curve to the recombination data and estimate genetic map positions of all considered markers. We note that this map is derived from an interspecific *A. lyrata* cross and may differ from the map of F1 hybrids. Mapping confidence intervals in particular, should therefore be interpreted cautiously.

If we include the possibility of sequencing or mapping errors at a uniform probability across all sites, E, then there are four ways in which we could sample a chromosome containing an allele, A, from site i. Similarly there are four ways that we could sample a chromosome containing the alternate allele, a (Table S1). These expression are therefore sufficient to evaluate the likelihood of a given value k at distorting site i by evaluating the likelihood of all mapped read counts across a chromosome from the male germline sequencing library.

Then, to estimate the effect size at a given site, p, we first used the somatic data from a single individual to estimate the proportion of reads derived from one parental species, *K*_s_, by using the likelihood of the possible sampling configurations (Table S1) to optimize this distortion parameter. *K*_s_ is now the null model against which we will test for evidence of distortion in the male germline sequencing library. We emphasize that an empirical null model is essential because it reflects much of the sequencing and mapping biases associated with each ancestry type and which would be challenging to model. We then evaluate the likelihood of the ratio *K*_s_ in our germline sample and optimize the germline‐derived read data to obtain *K*_g_. The relative likelihoods of the two ratios given the germline allele count data, *R*_g_, L(*k* = *K*_s_|*R*_g_) and L(*k* = *K*_g_|*R*_g_), then provides a straightforward means of evaluating the significance of the skew in ancestry at candidate distorting position i. Additionally, this approach can be used to identify the maximum likelihood estimate of the true distorting position and effect size by maximizing the likelihood ratio obtained across all possible positions along a chromosome. Software to perform this procedure is available from https://github.com/russcd/MAP_SD.

### CONSTRUCTING CONFIDENCE INTERVALS

In addition to providing a point estimate of a distorter's position, it is also important to quantity uncertainty in a distorter's estimated mapping position. We therefore implemented and evaluated a simple approach where we obtained confidence intervals for i by resampling read data by bootstrapping ancestry informative sites along a chromosome with replacement and rerunning our analysis. Functions to perform site and effect estimation as well as constructing distorter element confidence intervals are implemented within the software package.

To explore the properties of our proposed bootstrapping approach and to estimate our statistical power given our sequencing effort, we simulated k values of 0.505, 0.510, 0.520, 0.550, and 0.640 conditional on the total sequencing depth in somatic and germline libraries for individual one. These *k* values were selected to approximate rates we discovered in our sequence data. For each replicate set of simulations, we recorded the width and positions of the confidence interval as well as the maximum likelihood position estimate of the distorting element. For each value of *k*, we ran 100 replicates and performed 200 bootstraps for each replicate simulation. Scripts to generate simulated distorting read counts are provided in the GitHub repository.

Expression territories for *A. thaliana* orthologs as designated in Phytozome (https://phytozome.jgi.doe.gov/pz/portal.html) were determined from the Plant Ontology database (https://www.arabidopsis.org/tools/bulk/po/index.jsp). We used Gene Ontology functional annotation to determine whether genes in the identified intervals are involved in meiotic processes (GO:0051321), reproduction (GO:0000003), reproductive processes (GO:0022414), or any their 871 descendant GO terms.

#### Signatures of selection

To test for selective sweeps in the intervals identified, we used genomic resequencing data (Hämälä et al. 2018) from a population of *A. lyrata* that we chose from the same *A. lyrata* subspecies (*A. lyrata petraea*) as our *A. lyrata* parents. We aligned all short read data to the *A. lyrata* reference and recovered genotypes as described in (Corbett‐Detig et al. 2015a). We used SWEEPFINDER version 2 (DeGiorgio et al. 2016) under default conditions to scan for signatures of recent positive selection and we retained outliers for the composite likelihood ratio (CLR) statistic that were greater than all of the nearest CLR peaks within 50 Kb. We then retained only those CLR peaks within the predicted confidence interval for each of the putative segregation distortion loci that showed an excess of *A. lyrata* alleles.

## Results and Discussion

### SEQUENCING AND MAPPING

After mapping the parental short read data against the *A. lyrata* reference genome (Hu et al. 2011), genotyping and filtering, we obtained 852,400 sites that were differentially fixed between the two parental individuals and heterozygous in all F1 somatic and germline samples (Table S2). Hereafter these sites are referred to as ancestry informative sites.

### RAW ANCESTRY RATIOS

Reference bias remains an important issue for mapping short read data (Schneeberger et al. 2010; Corbett‐Detig and Hartl 2012; Paten et al. 2017), and is expected to be particularly problematic in our application because we mapped short read data to the *A. lyrata* reference genome that is much more closely related to the *A. lyrata* parents in each cross than to the *A. halleri* parents. We therefore expected that a substantially larger fraction of reads derived from *A. lyrata* chromosomes would map correctly than reads derived from *A. halleri* chromosomes. This consideration speaks to a key strength of our study's design: by including data from somatic tissues, we can establish the appropriate null model against which to test for evidence of segregation distortion.

**Figure 1:**
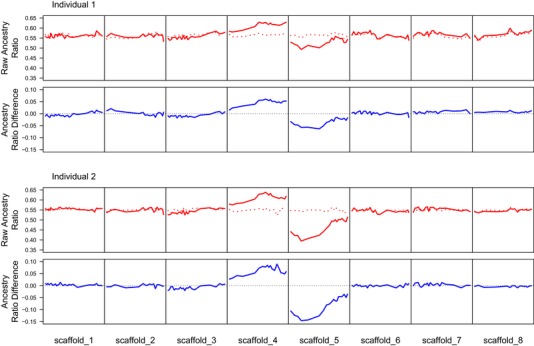
Raw ancestry ratios and differences between raw somatic and germline ancestry ratios in 1000 SNP nonoverlapping windows. Raw ancestry ratios of Individual one (top) and individual two (bottom) are shown with germline (solid red) and somatic (dashed red) in the first and third rows. The second and fourth rows show the differences between raw ancestry ratios for germline and somatic libraries in each window along the genome. The dotted horizontal line indicates the expected difference, 0, if the two ratios were exactly equal. Positive values indicate a bias toward excess *A. lyrata* ancestry and negative values indicate a bias toward excess *A. halleri* ancestry.

Consistent with this expectation, we observe skews in the ancestry ratio for both germline and somatic libraries toward an excess of *A. lyrata* alleles across the genome. Additionally, this variance is not uniform across the genome, but varies from window to window (Fig. 1). For five of the eight chromosomes, the differences in ancestry ratio between somatic and germline libraries (both overall and within individual windows) is slight, indicating little reason to suspect segregation distorters are present on these chromosomes. Furthermore, where we see difference in the ancestry ratio of raw read data on these chromosomes, it is rarely mirrored in the other individual. However, there are three chromosomes that show modest to large skews in ancestry ratios. On scaffolds 3, 4, and 5, we observe parallel differences where both pollen libraries consistently produce larger or smaller *A. lyrata* ancestry proportions, as do somatic libraries.

### POWER AND MAPPING PROPERTIES

To leverage the potentially diffuse signals of ancestry skews around segregation distortion loci, we developed a maximum likelihood approach for estimating the effect sizes and the map positions of distorting loci (see Methods). Briefly, our approach models the expected decay in the ancestry ratio around a distorting site as a function of the recombinational distance between the distorting locus and the read data along each chromosome. To evaluate the power of this approach given our sequencing efforts, we simulated distorting loci, conditional on the true distribution of ancestry informative sites and empirical read coverages, at randomly selected sites with ancestry ratio skews of 0.505, 0.510, 0.520, 0.550, and 0.640. We found that our approach can consistently identify distorting alleles with effect sizes 0.005 and greater, even when we apply a stringent *P*‐value cutoff to accommodate the inherent multiple testing challenges of our framework (e.g., uncorrected *P* <= 0.0005, Fig. S2). Similarly, the distance between the maximum likelihood position estimate and the true distorting locus decreases with increasing effect sizes. However, the error in our estimate of the distortion effect size, *k*, does not appear to change as a function of the true *k*.

When data is simulated under the assumed model of distortion, recombination, and sequencing, the false‐positive rate of our approach appears to be quite low. Specifically, across 100 replicates simulations with Mendelian segregation, we recovered a maximum *k* of 0.5034 and a maximum likelihood ratio of 9.17 (Fig. S3). Therefore, the false‐positive rate associated with our approach is relatively modest and unlikely to produce the large skews in ancestry ratios observed in real data when data are simulated under the assumed model of segregation distortion. However, we caution that unmodeled sources of read mapping variance such as somatic aneuploidy events (which may be especially common in hybrids, Huettel et al. 2008) or endoreduplication processes (whereby the nuclear genome is replicated to a large number of copies in the absence of cell division, especially in the leaf epidermal cells, Lee et al. 2009) might cause larger shifts in the estimation of k than we observe in these simple simulations… Therefore weak effect segregation distortion should be carefully scrutinized, using for example technical and biological replicates (see also below).

It is also valuable to quantify uncertainty in the estimated map positions of distorting genes. To do this, we propose a bootstrapping approach where ancestry informative sites along a given chromosome are resampled at random with replacement and the estimation procedure repeated. We note that this idea bears some similarity to commonly applied bootstrapping approaches for mapping confidence intervals in quantitative trait loci (Visscher et al. 1996). In applying this bootstrapping procedure to simulated datasets, we found that the 95% mapping confidence interval performs approximately as expected, except for very weak effect distorters (*k* < 0.505, Table S3). That is, the 95% confidence interval contains the true distorter position in approximately 95% of replicate simulations. Furthermore, the width of the confidence intervals is inversely related to *k*. Nonetheless, at larger *k* values, the confidence interval may be slightly conservative and our program tends to overestimate the width of the confidence interval in simulations. These results therefore suggest that a bootstrap resampling approach can be used to construct map confidence intervals around distorting loci.

### IDENTIFICATION AND FINE‐MAPPING SEGREGATION DISTORTION LOCI

**Figure 2:**
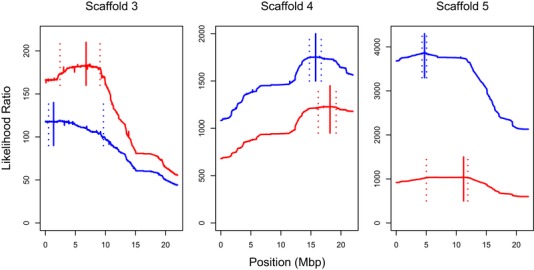
Estimated positions and confidence intervals of distorting loci for individual one (red) and individual two (blue). The likelihood ratio between the ancestry ratio estimated from somatic samples and the ancestry ratio estimate obtained from germline samples. The maximum likelihood map position is shown with solid vertical line and the 95% confidence interval, obtained from 1000 bootstrap replicates, is denoted using two dashed lines.

We discovered three sites that showed significant evidence of distortion in both individuals (Fig. 2). For all three cases, the direction of the ancestry skew in the pollen relative to the somatic sample is the same in both individuals, and for two loci the estimated effect sizes are very similar (Table S4). In agreement with the idea that the same distorting loci are acting in both individuals, we note that the 95% confidence intervals are overlapping for each locus. Collectively, these results are consistent with the presence of at least three moderate effect‐size segregation distortion loci across the hybrid genomes of these individuals.

Nonetheless, for one putative site of segregation distortion on Scaffold 5, our estimates of *k* are quite different between the two individuals (0.551 vs 0.631, Table S4). This might occur if the distortion effect of the same locus is different in the two individuals, perhaps due to additional polymorphic genetic modifiers (e.g., suppressors). This could also occur if an additional segregation distortion gene is present in only a single individual and is nearby on the same chromosome. It might be feasible to extend the maximum likelihood mapping framework that we developed above to accommodate and distinguish between single and two locus segregation distortion models if the two loci act independently. However, because we do not have a fine‐scale recombination map available for hybrids of these two species and because we do not know the molecular basis of segregation distortion, it would be premature to attempt to distinguish between single and two locus models.

We also note that the effect sizes that we measured are probably underestimates, since aborted pollen may still have contributed DNA to the pool collected in particular if abortion occurred at late stages of development and DNA in the aborted pollen was not entirely degraded.

### SMALL EFFECT SEGREGATION DISTORTION AND IMPACTS OF UNMODELED MAPPING VARIANCE

In addition to this set of three relatively large effect putative segregation distortion loci, we also recover ten candidate small‐effect segregation distorters on the other five chromosomes (Table S5). Although most distortion effects are nominally significant and exceed the largest *k*‐value we obtained from our null model based on Mendelian simulations (above), there are reasons to suspect other unmodeled sources of variation might be responsible rather than segregation distortion. First, for most loci, the 95% confidence intervals do not overlap between the two individuals. Second, those that do overlap tend to be quite large (e.g., more than ½ of the total length of the chromosome). Third, for four of the five chromosomes with evidence of weak effects, the estimated ancestry skew is in opposite directions in the two F1 individuals. It is therefore likely that additional factors influence the estimated ancestry ratios.

The plausible reasons for these apparent discrepancies are numerous. It is possible that subtle mapping biases affect read mapping of each library nonuniformly. Additionally, small‐scale somatic aneuploidy or segmental deletion and duplication events might actually cause the somatic sample ancestry ratio to skew from the expected 50:50 representation. We note that if such an event occurred in only a small proportion of cells in a sample, it could be very challenging to accurately distinguish between segregation distortion and somatic ancestry ratio distortion. We therefore cannot confidently exclude alternative explanations for these weak effect candidate segregation distorters, and we suggest that future efforts interested in accurately detecting and quantifying the impact of very weak segregation distorters may wish to produce large numbers of biological and technical replicates.

### MOLECULAR CAUSES AND RATE OF EVOLUTION OF SEGREGATION DISTORTION

It is important to estimate the rate at which segregation distortion loci arise between divergent lineages. Using an estimate of the divergence time between *A. lyrata* and *A. halleri*, 337,000 years (Roux et al. 2011), we therefore estimate that new segregation distortion loci arise and reach high frequencies in populations at an approximate rate of one per 224,000 years. The variance associated with this estimate is clearly quite large and might differ if these distorters were segregating within the ancestral population or are more likely to migrate between diverging populations with occasional gene flow. Additionally, as we have reduced confidence in our ability to accurately identify weak effect distorters, we cannot confidently distinguish weak biases from segregation distortion, and because some distorting genes may become nonfunctional after fixing within a lineage, ours is most likely an underestimate. Nonetheless, it is clear that the rate of evolution of distorters is relatively large.

There are at least two genetic mechanisms that might underlie segregation distortion in hybrid individuals. First, the independent evolution of selfish segregation distorter genes in each population (Lindholm et al. 2016). If there is little cost of the driver gene, they fix rapidly within populations (Hartl 1972), and can be unmasked in hybrids. Our results may therefore imply that the evolution of selfish elements is a fundamental contributor to the evolution of genomic differences among lineages. Second, rather than releasing selfish drivers in hybrid backgrounds, segregation distortion may also occur as a pathological response to hybridization (Coyne and Allen Orr 2004). Under such a model, distorting loci would be most similar to classical Dobzhansky‐Muller incompatibilities where a negative interaction between alleles fixed in either lineage results in incompatibilities within hybrid pollen where these previously untested alleles encounter each other. This might be particularly important in male germline tissue because genetic interactions within haploid genomes cannot be masked by dominant compatible alleles as in diploid tissues.

If the second model is driven by pairwise interactions of alleles from each parental population, each distorting locus should be matched with another that distorts in the direction of the other species’ allele. That is, in a purely pairwise model, interactions should manifest only in the pollen that inherits both alleles, and therefore we expect to find nearly identical and opposite ancestry skews immediately surrounding each site. That we observe this pattern in only one of four possible pairwise combinations of loci—scaffolds 4 and 5 in individual one may be close—suggests a pairwise haploid‐acting DMI model is insufficient to explain the bulk of our data. Rather, our results could be consistent with the evolution of three distinct selfish segregation distortion elements. However, it is possible that more complex multilocus incompatibilities or interactions between diploid and haploid acting genes exposed in hybrid pollen drive the observed segregation distortion. It may be feasible to distinguish some of these effects by applying similar methodology in advance intercross or backcross individuals and therefore additional research could help to illuminate the specific evolutionary origins of segregation distortion alleles acting in hybrid F1 pollen.

A simple test could use the framework developed here to resolve this question. If segregation distortion in pollen is due to gene interactions between diverging lineages, these effects should “snowball” and accumulate faster than linearly with divergence time (Orr 1995; Orr and Turelli 2001). Alternatively, if each instance of segregation distortion represents the evolution of an independent selfish gene, the accumulation of segregation distorters should be approximately linear with time. Therefore, by comparing the rate of occurrences across diverse hybrid individuals from populations with variable divergence times, it might be possible to distinguish these hypotheses by using the approach described here. This would involve expanding the present study to more pairs of closely related species.

### CANDIDATE SEGREGATION DISTORTION GENES

The intervals identified remain relatively large (from 0.50 to 7.19 Mb, Table S3) and contain a substantial number of genes, from 69 for the interval on chromosome 5 in individual two to 1762 for the one on chromosome 3 in individual one (Table S3). Assuming that both individuals contain identical distorters, we further reduced the intervals by focusing only on the genes in the overlapping portions of the confidence intervals obtained from the two individuals. Only 21 genes on scaffold 5 are retained when doing this, of which only seven are expressed in pollen (Table S6). Similarly, three of the genes in the interval on scaffold 3 have GO annotations related to meiotic or reproductive processes, including AL3G21800 (GO:0045132, meiotic chromosome segregation), AL3G24910 (GO:0007131, reciprocal meiotic recombination), and AL3G23610 (GO:0048544, recognition of pollen). At this step, it remains challenging to further explore the range of possible molecular mechanisms that might be causing those distortions, especially given the large proportion of genes expressed in pollen overall (Rutley and Twell 2015), the wide diversity of molecular functions potentially involved in SD (Lindholm et al. 2016) and also since detailed orthology maps between *A. halleri* and *A. lyrata*, including in particular the species‐specific genes would be required to compare the local genomic organization. Nonetheless, for the distorter on scaffolds 3 and 5, this highlights the ability of this method to identify a manageable number of genes for functional follow up work.

#### Signatures of selection

Given the relatively recent species divergence and potentially strong selective coefficients associated with the levels of segregation distortion observed, we then looked for evidence of recent selective sweeps in these regions. We used genomic resequencing data from a Swedish population of *A. lyrata* (Hämälä et al. 2018) and identified 19 and 7 genes with outlier composite likelihood ratios on scaffolds 3 and 5, respectively (Table S7), which are within the distorted regions on those chromosomes. Among those, AL3G18410 has a role in entry of microspores into mitosis and AL3G22840 is required for differentiation of microspores into pollen, making them prime candidates for the control of segregation distortion.

### QUANTIFYING THE CONTRIBUTION OF SEGREGATION DISTORTION TO REPRODUCTIVE ISOLATION

Regardless of the specific genetic mechanism of interaction, it is of interest to estimate the potential contribution of segregation distortion to reproductive isolation between *A. lyrata* and *A. halleri* populations. For example, if the skew in the ancestry ratio on Scaffold 3 for individual one is 0.011 in favor of *A. halleri* alleles, then the relative viability of pollen that inherits the *A. lyrata* allele at the distorting site on Scaffold 3 is 0.957 (i.e., by solving (1/(1+X)) = 0.511), resulting in a proportional decrease in the production across all viable pollen of 0.022. If each distorting locus acts independently, for example if all are unmasked independent segregation distortion genes, then these effects combine multiplicatively across each candidate locus to yield a pollen viability decrease of 0.2 and 0.33 for individuals one and two, respectively.

Alternatively, if combinations of parental alleles result in partially inviable pollen due to their interactions within haploid pollen cells, then each of the observed ancestry skews may not be independent and the proportion decrease in pollen viability will be smaller than if each locus acts separately. To estimate the minimum effect under this model, we use the pollen viability impact of the maximally distorted locus as a proxy to obtain an estimate of the minimum plausible impact of distorted segregation on pollen viability. For both individuals, the maximally distorted site is on Scaffold 5, and yields an estimate of the minimum pollen viability impact of segregation distortion of 0.1 and 0.21 for individuals one and two, respectively. We note that if some of the resources allotted to aborted pollen can be resorbed by the hybrid individual and repurposed to produce additional viable pollen, the total impact on male fertility in F1 individuals would be less than these estimates. Measuring pollen viability in these F1 hybrids using Alexander staining, for example, would be a way to evaluate the consequences of these incompatibilities. Decoupling the three distorter loci by additional crosses will now be required to dissect their individual contribution and determine whether they act independently (as expected in simple meiotic drive or haploid selection models) or interact in a complex fashion (as expected if they result from DMI).

### THE RELATIVE CONTRIBUTION OF GAMETIC TRANSMISSION DISTORTION

Substantial TRD has been studied previously for these species in both backcross (Willems et al. 2007) and F2 intercross (Willems et al. 2010) populations. Despite the limited power inherent to TRD, significant distortion was reported (nearly complete distortion at some loci across large chromosomal regions) in both studies. Importantly, many of the largest outliers for TRD in previous studies were found on chromosomes on which we do not detect distortion in pollen. This strongly suggests that additional genetic factors influence transmission in F1 hybrids or viability in later hybrid progeny beyond those reported here. In particular, we note that additional gametic factors may influence the growth of pollen or the probability a given pollen grain successfully fertilizes an ovule. Similarly, DMIs influencing viability will impact zygotic genotype representations. Unraveling the specific contributions of each stage could be achieved by recurrent sequencing of pooled populations at each developmental stage provided sufficient individuals can be recovered. Nonetheless, pollen viability effects act in early F1 reproduction and is therefore likely to be an important contributor to TRD within crosses among *A. lyrata* and *A. haleri*.

## Conclusion

Segregation distortion in pollen evolves rapidly between *Arabidopsis* lineages. Segregation distortion therefore has a substantial effect on F1 male transmission and may be an important contributor to reproductive isolation among *A. halleri* and *A. lyrata* populations when they encounter each other and hybridize in nature. More generally, because this approach simply and efficiently quantifies segregation distortion as well as maps distorting genes within hybrid male germline samples, it has the potential to enable substantially more quantitative and unbiased surveys of the prevalence and genetic basis of segregation distortion across diverse groups of organisms. These data are essential for resolving questions about the pace at which segregation distortion elements evolve within and between populations and the degree to which they contribute to the reproductive isolation of divergent taxa.

## DATA AND REAGENT AVAILABILITY

The short read data generated in this project are available through the sequence read archive under project accession number PRJNA503733. All software and scripts necessary to reproduce our results are available from the project github repository https://github.com/russcd/MAP_SD.

Associate Editor: S. Wright

## Supporting information


**Figure S1**. Empirical depth distribution among all sites that were found to be heterozygous in all F1 tissues and as fixed differences between all representatives of each parental species.

**Figure S2**. Statistical properties of the segregation distortion mapping approach.

**Figure S3**. Estimates of k and likelihood ratio for Mendelian simulations.

**Table S1**. Possible sampling configurations and their probabilities for alleles A and a.
**Table S2**. Crossing design, library Sequencing Yields and Index Information
**Table S3**. Performance of 100 bootstrapped estimates of the position of distorting loci in simulated datasets.
**Table S4**. Estimated mapping positions, confidence intervals, and effect sizes (k) for segregation distortion loci in individual one (above) and individual two (below). 859, 139 and 21 genes are contained in the overlapping intervals identified by the two individuals, among which 430, 52 and 7 are expressed in pollen (as determined in *A. thaliana*) for the distorters on scaffold 3, 4 and 5 respectively.
**Table S5**. Additional possible small‐effect segregation distortion loci on the remaining five chromosome arms.
**Table S6**. Genes expressed in pollen in the overlapping interval on scaffold 5
**Table S7**. Genomic positions with evidence for selective sweeps in *A. lyrata* in the intervals showing segregation distortion in scaffolds 3 and 5. For each 500bp window, the composite likelihood ratio (CLR) statistic compares the hypothesis of a complete selective sweep at the location to the null hypothesis of no sweep. For each significant position, the gene in which it is located or the nearest gene is reported, along with its *A. thaliana* ortholog (when present) and a brief annotation summary from TAIR. Expression in pollen of the *A. thaliana* ortholog is determined by https://www.arabidopsis.org/tools/bulk/po/

## References

[evl396-bib-0001] Bélanger, S. , I. Clermont , P. Esteves , and F. Belzile . 2016 Extent and overlap of segregation distortion regions in 12 barley crosses determined via a Pool‐GBS approach. Theor. Appl. Genet. 129:1393–1404. 27062517 10.1007/s00122-016-2711-5

[evl396-bib-0002] Bolger, A. M. , M. Lohse , and B. Usadel . 2014 Trimmomatic: a flexible trimmer for Illumina sequence data. Bioinformatics 30:2114–2120. 24695404 10.1093/bioinformatics/btu170 PMC4103590

[evl396-bib-0003] Burt, A. , and R. Trivers . 2009 Genes in conflict: The biology of selfish genetic elements. Harvard Univ. Press, Harvard.

[evl396-bib-0004] Castric, V. , J. Bechsgaard , M. H. Schierup , and X. Vekemans . 2008 Repeated adaptive introgression at a gene under multiallelic balancing selection. PLoS Genet. 4:e1000168. 18769722 10.1371/journal.pgen.1000168 PMC2517234

[evl396-bib-0005] Castric, V. , J. S. Bechsgaard , S. Grenier , R. Noureddine , M. H. Schierup , and X. Vekemans . 2010 Molecular evolution within and between self‐incompatibility specificities. Mol. Biol. Evol. 27:11–20. 19773365 10.1093/molbev/msp224

[evl396-bib-0006] Corbett‐Detig, R. B. , and D. L. Hartl . 2012 Population genomics of inversion polymorphisms in *Drosophila melanogaster* . PLoS Genet. 8:e1003056. 23284285 10.1371/journal.pgen.1003056 PMC3527211

[evl396-bib-0007] Corbett‐Detig, R. B. , D. L. Hartl , and T. B. Sackton . 2015a Natural selection constrains neutral diversity across a wide range of species. PLoS Biol. 13:e1002112. 25859758 10.1371/journal.pbio.1002112 PMC4393120

[evl396-bib-0008] Corbett‐Detig, R. , E. Jacobs‐Palmer , D. Hartl , and H. Hoekstra . 2015b Direct gamete sequencing reveals no evidence for segregation distortion in house mouse hybrids. PLoS One 10:e0131933. 26121240 10.1371/journal.pone.0131933 PMC4487504

[evl396-bib-0009] Coyne, J. A. , and H. Allen Orr . 2004 Speciation. Sinauer Associates Incorporated, Sunderland, Massachusetts.

[evl396-bib-0010] DeGiorgio, M. , C. D. Huber , M. J. Hubisz , I. Hellmann , and R. Nielsen . 2016 SweepFinder2: increased sensitivity, robustness and flexibility. Bioinformatics 32:1895–1897. 27153702 10.1093/bioinformatics/btw051

[evl396-bib-0011] de Meaux, J. , A. Pop , and T. Mitchell‐Olds . 2006 Cis‐regulatory evolution of chalcone‐synthase expression in the genus *Arabidopsis* . Genetics 174:2181–2202. 17028316 10.1534/genetics.106.064543 PMC1698642

[evl396-bib-0012] DePristo, M. A. , E. Banks , R. Poplin , K. V. Garimella , J. R. Maguire , C. Hartl , A. A. Philippakis , G. del Angel , M. A. Rivas , M. Hanna , et al. 2011 A framework for variation discovery and genotyping using next‐generation DNA sequencing data. Nat. Genet. 43:491–498. 21478889 10.1038/ng.806 PMC3083463

[evl396-bib-0013] Fishman, L. , and J. H. Willis . 2005 A novel meiotic drive locus almost completely distorts segregation in mimulus (monkeyflower) hybrids. Genetics 169:347–353. 15466426 10.1534/genetics.104.032789 PMC1448871

[evl396-bib-0014] Frérot, H. , M.‐P. Faucon , G. Willems , C. Godé , A. Courseaux , A. Darracq , N. Verbruggen , and P. Saumitou‐Laprade . 2010 Genetic architecture of zinc hyperaccumulation in *Arabidopsis halleri*: the essential role of QTL x environment interactions. New Phytol. 187:355–367. 20487314 10.1111/j.1469-8137.2010.03295.x

[evl396-bib-0015] Frérot, H. , N.‐C. Hautekèete , I. Decombeix , M.‐H. Bouchet , A. Créach , P. Saumitou‐Laprade , Y. Piquot , and M. Pauwels . 2017 Habitat heterogeneity in the pseudometallophyte *Arabidopsis halleri* and its structuring effect on natural variation of zinc and cadmium hyperaccumulation. Plant Soil 423:157–174.

[evl396-bib-0016] Hämälä, T. , T. M. Mattila , P. H. Leinonen , H. Kuittinen , and O. Savolainen . 2017 Role of seed germination in adaptation and reproductive isolation in *Arabidopsis lyrata* . Mol. Ecol. 26:3484–3496. 28393414 10.1111/mec.14135

[evl396-bib-0017] Hämälä, T. , T. M. Mattila , and O. Savolainen . 2018 Local adaptation and ecological differentiation under selection, migration, and drift in *Arabidopsis lyrata* ^*^ . Evolution 72:1373–1386. 10.1111/evo.13502 29741234

[evl396-bib-0018] Hartl, D. L. 1972 Population dynamics of sperm and pollen killers. Theor. Appl. Genet. 42:81–88. 24430773 10.1007/BF00277948

[evl396-bib-0019] Huettel, B. , D. P. Kreil , M. Matzke , and A. J. M. Matzke . 2008 Effects of aneuploidy on genome structure, expression, and interphase organization in *Arabidopsis thaliana* . PLoS Genet. 4:e1000226. 18927630 10.1371/journal.pgen.1000226 PMC2562519

[evl396-bib-0020] Hu, T. T. , P. Pattyn , E. G. Bakker , J. Cao , J.‐F. Cheng , R. M. Clark , N. Fahlgren , J. A. Fawcett , J. Grimwood , H. Gundlach , et al. 2011 The *Arabidopsis lyrata* genome sequence and the basis of rapid genome size change. Nat. Genet. 43:476–481. 21478890 10.1038/ng.807 PMC3083492

[evl396-bib-0021] Larson, E. L. , D. Vanderpool , B. A. J. Sarver , C. Callahan , S. Keeble , L. P. Provencio , M. D. Kessler , V. Stewart , E. Nordquist , M. D. Dean , et al. 2018 The evolution of polymorphic hybrid incompatibilities in house mice. Genetics 209:845–859. 29692350 10.1534/genetics.118.300840 PMC6028243

[evl396-bib-0022] Lee, H. O. , J. M. Davidson , and R. J. Duronio . 2009 Endoreplication: polyploidy with purpose. Genes Dev. 23:2461–2477. 19884253 10.1101/gad.1829209 PMC2779750

[evl396-bib-0023] Leppala, J. , F. Bokma , and O. Savolainen . 2013 Investigating incipient speciation in *Arabidopsis lyrata* from patterns of transmission ratio distortion. Genetics 194:697–708. 23666938 10.1534/genetics.113.152561 PMC3697974

[evl396-bib-0024] Li, H. , and R. Durbin . 2009 Fast and accurate short read alignment with Burrows‐Wheeler transform. Bioinformatics 25:1754–1760. 19451168 10.1093/bioinformatics/btp324 PMC2705234

[evl396-bib-0025] Lindholm, A. K. , K. A. Dyer , R. C. Firman , L. Fishman , W. Forstmeier , L. Holman , H. Johannesson , U. Knief , H. Kokko , A. M. Larracuente , et al. 2016 The ecology and evolutionary dynamics of meiotic drive. Trends Ecol. Evol. 31:315–326. 26920473 10.1016/j.tree.2016.02.001

[evl396-bib-0026] Macnair, M. R. , V. Bert , S. B. Huitson , P. Saumitou‐Laprade , and D. Petit . 1999 Zinc tolerance and hyperaccumulation are genetically independent characters. Proc. Biol. Sci. 266:2175–2179. 10649632 10.1098/rspb.1999.0905 PMC1690342

[evl396-bib-0027] McKenna, A. , M. Hanna , E. Banks , A. Sivachenko , K. Cibulskis , A. Kernytsky , K. Garimella , D. Altshuler , S. Gabriel , M. Daly , et al. 2010 The genome analysis toolkit: a MapReduce framework for analyzing next‐generation DNA sequencing data. Genome Res. 20:1297–1303. 20644199 10.1101/gr.107524.110 PMC2928508

[evl396-bib-0028] Novikova, P. Y. , N. Hohmann , V. Nizhynska , T. Tsuchimatsu , J. Ali , G. Muir , A. Guggisberg , T. Paape , K. Schmid , O. M. Fedorenko , et al. 2016 Sequencing of the genus Arabidopsis identifies a complex history of nonbifurcating speciation and abundant trans‐specific polymorphism. Nat. Genet. 48:1077–1082. 27428747 10.1038/ng.3617

[evl396-bib-0029] Novitski, E. , Y. Hirazumi , and E. Matsunaga . 1962 Meiotic drive. Science 137:861–862. 14480613

[evl396-bib-0030] Orr, H. A. 1995 The population genetics of speciation: the evolution of hybrid incompatibilities. Genetics 139:1805–1813. 7789779 10.1093/genetics/139.4.1805 PMC1206504

[evl396-bib-0031] Orr, H. A. , and M. Turelli . 2001 The evolution of postzygotic isolation: accumulating Dobzhansky‐Muller incompatibilities. Evolution 55:1085–1094. 11475044 10.1111/j.0014-3820.2001.tb00628.x

[evl396-bib-0032] Paten, B. , A. M. Novak , J. M. Eizenga , and E. Garrison . 2017 Genome graphs and the evolution of genome inference. Genome Res. 27:665–676. 28360232 10.1101/gr.214155.116 PMC5411762

[evl396-bib-0033] Phadnis, N. , and H. A. Orr . 2009 A single gene causes both male sterility and segregation distortion in Drosophila hybrids. Science 323:376–379. 19074311 10.1126/science.1163934 PMC2628965

[evl396-bib-0034] Roux, C. , V. Castric , M. Pauwels , S. I. Wright , P. Saumitou‐Laprade , and X. Vekemans . 2011 Does speciation between *Arabidopsis halleri* and *Arabidopsis lyrata* coincide with major changes in a molecular target of adaptation? PLoS One 6:e26872. 22069475 10.1371/journal.pone.0026872 PMC3206069

[evl396-bib-0035] Rutley, N. , and D. Twell . 2015 A decade of pollen transcriptomics. Plant Reprod. 28:73–89. 25761645 10.1007/s00497-015-0261-7 PMC4432081

[evl396-bib-0036] Sandler, L. , and E. Novitski . 1957 Meiotic drive as an evolutionary force. Am. Nat. 91:105–110.

[evl396-bib-0037] Schneeberger, K. , S. Ossowski , F. Ott , J. D. Klein , X. Wang , C. Lanz , L. M. Smith , J. Cao , J. Fitz , N. Warthmann , et al. 2010 Reference‐guided assembly of four diverse *Arabidopsis thaliana* genomes. Proc. Natl. Acad. Sci. USA 108:10249–10254. 10.1073/pnas.1107739108 PMC3121819 21646520

[evl396-bib-0038] Taylor, D. R. , and P. K. Ingvarsson . 2003 Common features of segregation distortion in plants and animals. Genetica 117:27–35. 12656570 10.1023/a:1022308414864

[evl396-bib-0039] Visscher, P. M. , R. Thompson , and C. S. Haley . 1996 Confidence intervals in QTL mapping by bootstrapping. Genetics 143:1013–1020. 8725246 10.1093/genetics/143.2.1013 PMC1207319

[evl396-bib-0040] Wang, W.‐K. , C.‐W. Ho , K.‐H. Hung , K.‐H. Wang , C.‐C. Huang , H. Araki , C.‐C. Hwang , T.‐W. Hsu , N. Osada , and T.‐Y. Chiang . 2010 Multilocus analysis of genetic divergence between outcrossing Arabidopsis species: evidence of genome‐wide admixture. New Phytol. 188:488–500. 20673288 10.1111/j.1469-8137.2010.03383.x

[evl396-bib-0041] Wei, K. H.‐C. , H. M. Reddy , C. Rathnam , J. Lee , D. Lin , S. Ji , J. M. Mason , A. G. Clark , and D. A. Barbash . 2017 A pooled sequencing approach identifies a candidate meiotic driver in *Drosophila* . Genetics 206:451–465. 28258181 10.1534/genetics.116.197335 PMC5419488

[evl396-bib-0042] Willems, G. , D. B. Dräger , M. Courbot , C. Godé , N. Verbruggen , and P. Saumitou‐Laprade . 2007 The genetic basis of zinc tolerance in the metallophyte *Arabidopsis halleri* ssp. halleri (Brassicaceae): an analysis of quantitative trait loci. Genetics 176:659–674. 17409091 10.1534/genetics.106.064485 PMC1893047

[evl396-bib-0043] Willems, G. , H. Frérot , J. Gennen , P. Salis , P. Saumitou‐Laprade , and N. Verbruggen . 2010 Quantitative trait loci analysis of mineral element concentrations in an *Arabidopsis halleri* × *Arabidopsis lyrata* petraea F2 progeny grown on cadmium‐contaminated soil. New Phytol. 187:368–379. 20487315 10.1111/j.1469-8137.2010.03294.x

