# Supplementary figures and images for: Bulk pollen sequencing reveals rapid evolution of segregation distortion in the male germline of Arabidopsis hybrids
Source: Evol Lett. 2019 Jan 30;3(1):93–103. doi: 10.1002/evl3.96 (PMC6369960; doi:10.1002/evl3.96)

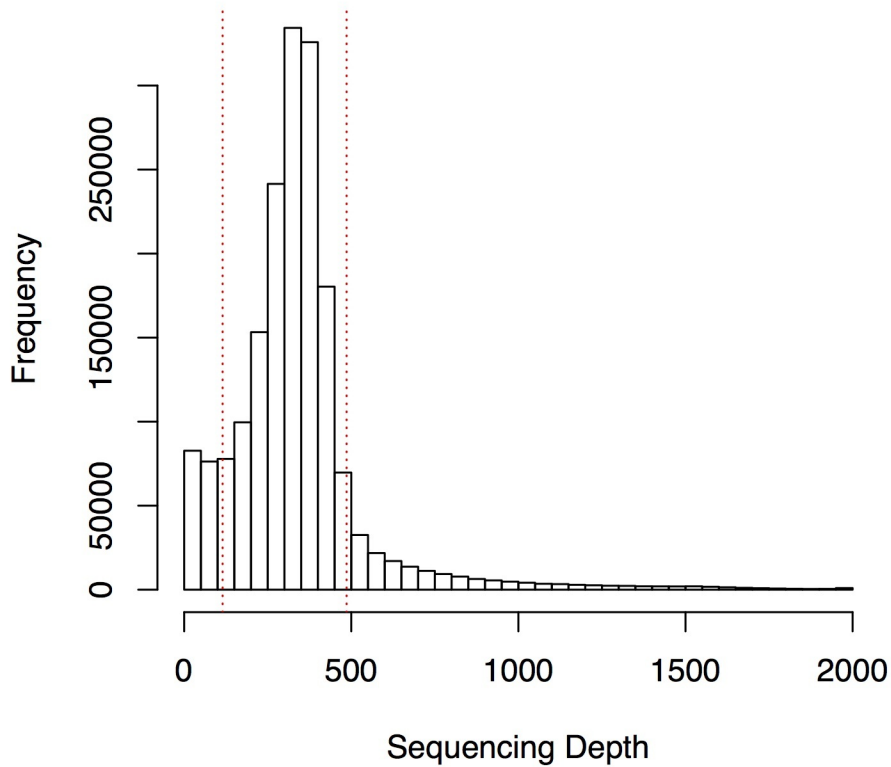

Supplement: Supplementary file 1 — Figure S1. Empirical depth distribution among all sites that were found to be heterozygous in all F1 tissues and as fixed differences between all representatives of each parental species. [file EVL3-3-93-s001.pdf]

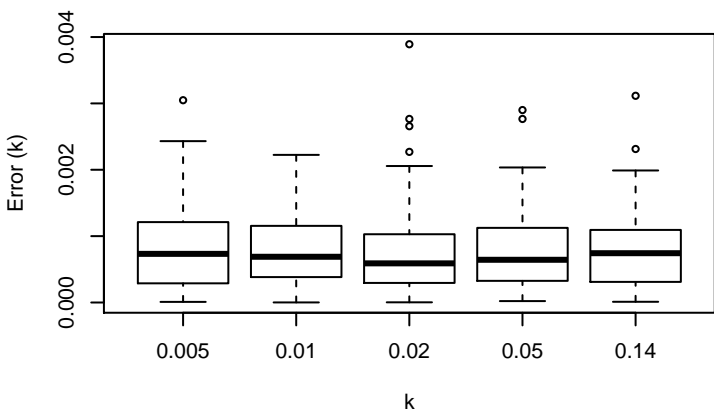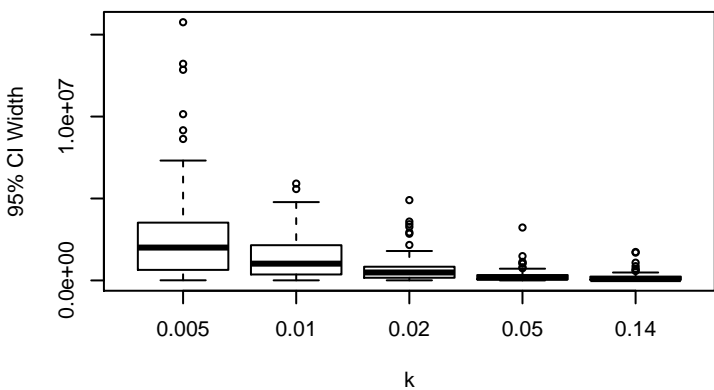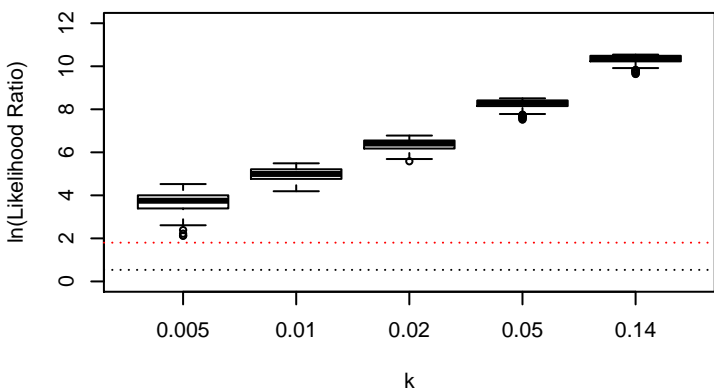

Supplement: Supplementary file 2 — Figure S2. Statistical properties of the segregation distortion mapping approach. [file EVL3-3-93-s002.pdf]

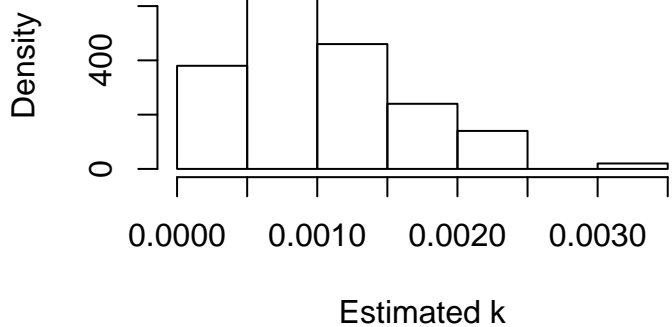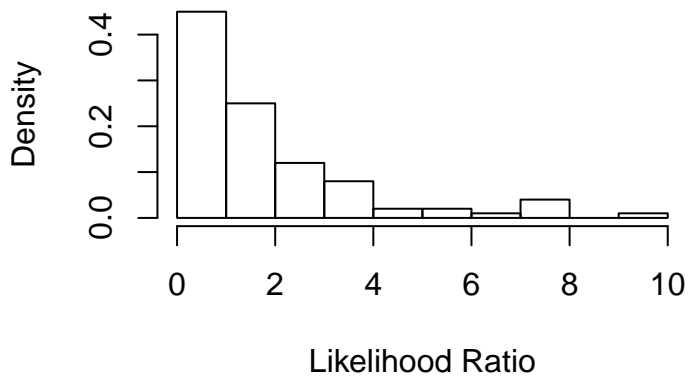

Supplement: Supplementary file 3 — Figure S3. Estimates of k and likelihood ratio for Mendelian simulations. [file EVL3-3-93-s003.pdf]
