# Supplementary material for: Bulk pollen sequencing reveals rapid evolution of segregation distortion in the male germline of Arabidopsis hybrids
Source: Evol Lett. 2019 Jan 30;3(1):93–103. doi: 10.1002/evl3.96 (PMC6369960; doi:10.1002/evl3.96)
Supplement: Supplementary file 4 — Table S1. Possible sampling configurations and their probabilities for alleles A and a. Table S2. Crossing design, library Sequencing Yields and Index Information Table S3. Performance of 100 bootstrapped estimates of the position of distorting loci in simulated datasets. Table S4. Estimated mapping positions, confidence intervals, and effect sizes (k) for segregation distortion loci in individual one (above) and individual two (below). 859, 139 and 21 genes are contained in the overlapping intervals identified by the two individuals, among which 430, 52 and 7 are expressed in pollen (as determined in A. thaliana) for the distorters on scaffold 3, 4 and 5 respectively. Table S5. Additional possible small‐effect segregation distortion loci on the remaining five chromosome arms. Table S6. Genes expressed in pollen in the overlapping interval on scaffold 5 Table S7. Genomic positions with evidence for selective sweeps in A. lyrata in the intervals showing segregation distortion in scaffolds 3 and 5. For each 500bp window, the composite likelihood ratio (CLR) statistic compares the hypothesis of a complete selective sweep at the location to the null hypothesis of no sweep. For each significant position, the gene in which it is located or the nearest gene is reported, along with its A. thaliana ortholog (when present) and a brief annotation summary from TAIR. Expression in pollen of the A. thaliana ortholog is determined by https://www.arabidopsis.org/tools/bulk/po/ [file EVL3-3-93-s004.docx]

**Table S1. Possible sampling configurations and their probabilities for alleles A and a.**

| Allele | Read Configuration | Probability |
| --- | --- | --- |
| A | (1) sampled from a distorting chromosome, there was no recombination and there was no sequencing error | p(A1) = k*(1-r_ip_)*(1-E) |
| A | (2) sampled from a non-distorting chromosome, there was a recombination event and there was no sequencing error | p(A2) = (1-k)*r_ip_*(1-E) |
| A | (3) sampled from a non-distorting chromosome, there was no recombination and there was a sequencing error | p(A3) = (1-k)*(1-r_ip_)*E |
| A | (4) sampled from a distorting chromosome, there was a recombination event and there was a sequencing error | p(A4) = k*r_ip_*E |
| a | (1) sampled from a non-distorting chromosome, there was no recombination and there was no sequencing error | p(a1) = (1-k)*(1-r_ip_)*(1-E) |
| a | (2) sampled from distorting chromosome, there was a recombination event and there was no sequencing error | p(a2) = k*r_ip_*(1-E) |
| a | (3) sampled from a distorting chromosome, there was no recombination and there was a sequencing error | p(a3) = k*(1-r_ip_)*E |
| a | (4) sampled from a non-distorting chromosome, there was a recombination event and there was a sequencing error | p(a4) = (1-k)*r_ip_*E |

**Table S2. Crossing design, library Sequencing Yields and Index Information**

| Type |  | tissue | ID | Yield (Reads) | I7 Index | I5 Index |
| --- | --- | --- | --- | --- | --- | --- |
| *A. lyrata* | Female parent | leaf | CP1-1 | 186714352 | AGGCAGAA | AGAGTAGA |
| *A. halleri* | Male parent | leaf | I14-16 | 187971076 | TAGGCATG | AGAGTAGA |
| Individual 2 | F1 | leaf | F65-19 | 421627172 | TAAGGCGA | TATCCTCT |
| Individual 2 | F1 | Bulk pollen | P65-19 | 497942260 | GGACTCCT | TATCCTCT |
| *A. lyrata* | Female parent | leaf | CP99-1-9 | 135128000 | TAAGGCGA | AGAGTAGA |
| *A. halleri* | Male parent | leaf | I16-8 | 454120500 | GGACTCCT | AGAGTAGA |
| Individual 1 | F1 | leaf | F63-8 | 388826880 | AGGCAGAA | TATCCTCT |
| Individual 1 | F1 | Bulk pollen | P63-8 | 447916956 | TAGGCATG | TATCCTCT |

**Table S3 Performance of 100 bootstrapped estimates of the position of distorting loci in simulated datasets.**

| k | Proportion of simulations outside of 95% CI | Mean 95% CI Width (Mb) |
| --- | --- | --- |
| 0.505 | 0.08 | 12.75 |
| 0.51 | 0.06 | 6.50 |
| 0.52 | 0.05 | 3.54 |
| 0.55 | 0.04 | 1.35 |
| 0.64 | 0.02 | 0.45 |

**Table S4. Estimated mapping positions, confidence intervals, and effect sizes (k) for segregation distortion loci in individual one (above) and individual two (below). 859, 139 and 21 genes are contained in the overlapping intervals identified by the two individuals, among which 430, 52 and 7 are expressed in pollen (as determined in *A. thaliana*) for the distorters on scaffold 3, 4 and 5 respectively.**

|  | Contig | Position (bp) | 95% Confidence Interval | k | Number of genes in the CI | Genes expressed in pollen |
| --- | --- | --- | --- | --- | --- | --- |
| Individual one | Scaffold 3 | 4,387,500 | 2,094,217-9,289,544 | 0.511 | 1,762 | 827 |
|  | Scaffold 4 | 16,616,280 | 15,616,938-16,975,205 | 0.447 | 310 | 121 |
|  | Scaffold 5 | 5,126,306 | 4,837,859-5,593,923 | 0.551 | 83 | 30 |
| Individual two | Scaffold 3 | 3,260,525 | 523,583-5,471,649 | 0.513 | 1,267 | 614 |
|  | Scaffold 4 | 15,347,370 | 15,027,487-16,257,312 | 0.426 | 275 | 98 |
|  | Scaffold 5 | 4,629,367 | 4,454,303-4,959,754 | 0.631 | 69 | 14 |

**Table S5. Additional possible small-effect segregation distortion loci on the remaining five chromosome arms.**

|  | Chromosome | Position | CI Start | CI End | k | Likelihood Ratio |
| --- | --- | --- | --- | --- | --- | --- |
| Individual one | scaffold_1 | 4969744 | 441741 | 10103223 | 0.506 | 221.566 |
|  | scaffold_2 | 103489 | 46845 | 16497935 | 0.493 | 43.187 |
|  | scaffold_6 | 3945724 | 2876456 | 5420462 | 0.495 | 78.685 |
|  | scaffold_7 | 14050705 | 13009871 | 17716235 | 0.493 | 240.053 |
|  | scaffold_8 | 14473353 | 13885464 | 20095287 | 0.492 | 177.578 |
|  |  |  |  |  |  |  |
| Individual two | scaffold_1 | 1794950 | 78584 | 6344720 | 0.494 | 106.259 |
|  | scaffold_2 | 921287 | 483660 | 18929493 | 0.502 | 3.6522 |
|  | scaffold_6 | 13048760 | 11274418 | 19514172 | 0.502 | 11.174 |
|  | scaffold_7 | 7025664 | 5833007 | 7720837 | 0.495 | 76.078 |
|  | scaffold_8 | 4336161 | 1403339 | 10910417 | 0.505 | 34.8624 |

**Table S6. Genes expressed in pollen in the overlapping interval on scaffold 5**

| 1. ***lyrata gene*** | 1. ***thaliana ortholog*** | ***A. thaliana* ortholog annotation** |
| --- | --- | --- |
| AL5G18320 | AT3G28690 | Protein kinase |
| AL5G18340 | AT3G28700 | Protein arginine methyltransferase |
| AL5G18480 | AT3G28750 | Uncharacterized protein |
| AL5G18440 | AT3G28720 | Transmembrane protein |
| AL5G18450 | AT3G28730 | Nucleosome/Chromatin assembly factor D |
| AL5G18460 | AT3G28740 | Member of the cytochrome p450 family |
| AL5G18520 | AT3G28780 | transmembrane protein, putative (DUF1216) |

**Table S7. Genomic positions with evidence for selective sweeps in *A. lyrata* in the intervals showing segregation distortion in scaffolds 3 and 5. For each 500bp window, the composite likelihood ratio (CLR) statistic compares the hypothesis of a complete selective sweep at the location to the null hypothesis of no sweep. For each significant position, the gene in which it is located or the nearest gene is reported, along with its *A. thaliana* ortholog (when present) and a brief annotation summary from TAIR. Expression in pollen of the *A. thaliana* ortholog is determined by https://www.arabidopsis.org/tools/bulk/po/**

| **Scaffold** | **Position** | **CLR** | **A. lyrata gene** | **A. thaliana gene** | **Annotation** | Expressed in pollen? |
| --- | --- | --- | --- | --- | --- | --- |
| Scaffold 3 | 2,168,603 | 27.0 | AL3G16330 | AT3G05675 | Protein ubiquination | Y |
|  | 2,383,607 | 28.2 | AL3G16840 | AT3G06020 | Regulation of mesistem growth | N |
|  | 2,479,608 | 31.6 | AL3G17090 | AT3G06230 | Activation of protein kinase activity | NA |
|  | 2,532,109 | 29.5 | AL3G17240 | AT3G06310 | Mitochondrial electron transport | Y |
|  | 2,746,612 | 57.4 | AL3G17660 | AT3G06610 | DNA binding | Y |
|  | 2,880,114 | 25.1 | AL3G18030 | AT3G06910 | Protein desumoylation, proteolysis | Y |
|  | 2,950,615 | 39.8 | AL3G18220 | AT3G07010 | Pectin catabolic process | Y |
|  | 3,014,116 | 28.7 | AL3G18410 | AT3G07160 | Has a role in entry of microspores into mitosis | Y |
|  | 3,338,121 | 26.4 | AL3G19370 | AT3G07990 | Proteolysis | Y |
|  | 3,550,624 | 25.5 | AL3G19990 | AT3G08970 | Cellular response to heat, pollen tube growth, protein folding, response to heat. At high temperatures, mutation alleles are not transmitted through the pollen due to defects in pollen tube growth | Y |
|  | 3,737,627 | 31.0 | AL3G20570 | AT3G09400 | Protein dephosphorylation | NA |
|  | 3,846,129 | 25.8 | AL3G20940 | AT3G09720 | P-loop containing nucleoside triphosphate hydrolases superfamily | N |
|  | 4,028,631 | 37.1 | AL3G21430 | AT3G10090 | Structural constituent of ribosome | Y |
|  | 4,174,633 | 26.5 | AL3G21810 | AT3G10450 | Proteolysis, secondary metabolic process | N |
|  | 4,322,636 | 34.5 | AL3G22250 | NA | NA | NA |
|  | 4,536,639 | 28.5 | AL3G22840 | AT3G11330 | Microgametogenesis, pollen development, required for differentiation of microspores into pollen | N |
|  | 4,813,643 | 35.7 | AL3G23540 | AT3G11945 | Carotenoid biosynthetic process | Y |
|  | 5,063,647 | 27.7 | AL3G24330 | AT3G12660 | Petal differentiation and expansion stage | Y |
|  | 5,145,148 | 53.1 | AL3G24530 | AT3G12860 | SnoRNA binding | N |
|  |  |  |  |  |  |  |
| Scaffold 5 | 4,856,768 | 37.5 | AL5G18350 | NA | NA | NA |
|  | 5,066,773 | 28.2 | AL5G18630 | AT3G28860 | Encodes a member of the ATP-binding cassette (ABC) Transporter family that is involved in auxin transport. | Y |
|  | 5,118,774 | 28.7 | AL5G18650 | NA | NA | NA |
|  | 5,214,777 | 61.4 | AL5G18710 | AT3G28920 | Glucosinolate metabolic proces, DNA binding transcription factor. | Y |
|  | 5,320,280 | 26.5 | AL5G18840 | AT3G28980 | Mediator of RNA Polymerase II transcription subunit-like protein | Y |
|  | 5,386,281 | 25.8 | AL5G18920 | AT3G29040 | Cysteine-rich repeat secretory protein | NA |
|  | 5,543,285 | 30.7 | AL5G19130 | NA | NA | NA |
